# The History and Capabilities of Herbal Simples

**Published:** 1890-09-20

**Authors:** W. T. Fernie


### The History and Capabilities of Herbal Simples.

By W. T. Fernie, M.D.

*(Continued from page 356.)*

### XVIII.—CHERVIL—CELANDINE.

### Chervil.

"Touching the King"—said the sweet Psalmist of Israel—"all thy garments smell of myrrh, aloes, and cassia from the ivory palaces—God hath anointed thee with the oil of gladness above thy fellows." For promoting which latter supreme purpose the fragrant herb Chervil would have been richer in cordial qualities than all the grass of the field. Its botanical name, *chærophyllum*, means *phyllum*, a plant, which, *kairei*, rejoices the heart. The roots, said an ancient writer, are "very good for old people that are dull and without courage; they gladden and comfort the spirits, and do increase their lusty strength." Former physicians held this plant in high esteem as capable of curing most chronic disorders, particularly with regard to the urinary passages and the gravel. Some even asserted that if these distempers would not yield to a constant use of chervil, they were scarcely curable by any other medicine. In modern times the plant has taken rank as a pot herb in our gardens, though its virtues and uses are not widely known. "There is found," writes Parkinson, "during June and July, in almost every English hedge a certain plant called Chærophyllum, in show very like unto Hemlockes, of a good and pleasant smell and taste, which have caused us to term it Sweet Chervill. The root is great, thick, and long; exceeding sweet in smell, and tasting like unto anise seeds. This same is used much among the Dutch people in a kind of loblolly or hotchpot, which they do eat—called warmus. The seeds taken as a salad, whilst they are yet green, exceed all other salads by many degrees in pleasantness of taste, sweetness of smell, and wholesomeness for the cold, and feeble stomach." Wild chervil will help to dissolve any tumors or swelling in all parts of the body speedily if applied to the place, as also to take away the spots, and marks in the flesh and skin of congealed blood by bruises or blows. In common with other camphoraceous and strongly aromatic herbs, by reason of its volatile oil and its terebenthine properties, the scandix, or sweet chervil was therefore truly entitled to make one of the choice spices used for composing the holy oil with which the sacred vessels of the tabernacle were anointed by Moses. It belongs to the particular group of umbelliferous plants which is endowed with balsamic gums, and with carminative essences appealing powerfully to the sense of smell. Catullus, of Roman days, may have had this herb in his mind when discoursing soft verse to his friend Fabullus:

"Nam unguentum dabo quod meæ puellæ

Donarunt, Veneres, Cupidines que;

Quod tu, quum olfacies, deo rogabis

Totum ut te faciat, Fabulle, nasum."

"I will give you a perfume my damsels gave me,

Sweet daughters of Venus, sad hoydens are ye!

Which the moment you smell will incite you to pray,

My Fabullus, to live as 'all nose' from that day."

"The great sweet cherville," said Gerard (1597) "groweth in my garden, and in the gardens of other men who have been diligent in these matters." Evelyn (1565) taught that "the tender tops of chervile should never be wanting in our sallets, being exceeding wholesome and chearing the spirits; also that the roots boiled and cold are to be much commended for aged persons." Furthermore, says he, the plant is used in tarts like spinach, and serves alone for divers sauces. In 1745 several Dutch soldiers were poisoned by eating the rough wild chervil, from which the cultivated sweet variety is distinguished by having its stems swollen beneath the joints—much as our patricians are signalised by gouty knuckles and toes. The feathery leaves of chervil, which are of a bright emerald hue in the spring, become of a rich purple in the autumn, just as the objectionably carroty locks of Tittlebat Titmouse, in *Ten Thousand a-Year*, became vividly green under Cyanochaitanthropopoion, and were afterwards strangely empurpled by Tetragmenon Abracadabra, at nine and sixpence the bottle. The juice of the herb is slightly aperient, and abundantly lactiferous when mixed with goat's milk or taken in gruel.

### Celandine: The Greater.

In the forty-sixth year of her age, the lion-hearted Queen Elizabeth was attacked with such grievous toothache that she could obtain no rest by night or day, because of the torture she endured. The lords of her council decided on sending for an "outlandish physician," named Penatus, who was famous for curing this agonising pain. He advised that, when all was said and done, if the tooth were hollow, it were best to have it drawn; but as Her Majesty could not bring herself to submit to the use of chirurgical instruments, he suggested that the juice of Chelidonius major—our larger Celandine—should be put into the tooth, and this stopped with wax, which would so loosen the tooth that in a short time it might be pulled out with the fingers. Aylmer, Bishop of London, tried to encourage the Queen by telling her that, though he was an old man and had not many teeth to spare, she could see a practical experiment made on himself. Thereupon he bade the surgeon who was in attendance extract one of his teeth in Her Majesty's presence. This plant, the larger Celandine, now called Chelidonium majus, and belonging to the poppy tribe, is still used in Suffolk for toothache by way of fomentation. It goes likewise by the name of fenugreek; fænum Græcum, Grecian hay; and by that of "tetterwort." The botanical name, Chelidonium, is a corruption of the Greek *chelidon*, a swallow; from an ancient tradition that the bird used this plant to restore the sight of its young:—

"Cœcatis pullis hac lumina mater hirundo,
Plinius ut scripsit—quamvis sint eruta, reddit."

It grows freely in our waste places and hedgerows, and may be readily known by the bright yellow juice which it exudes when bruised. On the doctrine of signatures this was formerly supposed to be curative of jaundice; and a medicinal tincture made from the entire plant is at the present time held in high esteem by many physicians for relieving torpid states of the liver. Eight or ten drops of this tincture, or of the fresh juice of the plant, may be given for a dose three times a day, in sweetened water, when there is bilious yellowness of the skin, with itching, and with clayey stools, dark, thick urine, constipation, and a pain in the right shoulder; also for neuralgia of the head and face on the right side. The root of this plant contains chemically chelidonine and sanguinarine. Clusius found by experience that the juice of the great celandine, when squeezed into small green wounds of what sort soever, wonderfully cured them. If the juice to the bigness of a pin's head be dropped into the eye in the morning, in bed, it takes away outward specks, and stops beginning suffusions. Also, if the yellow juice is applied to warts, first gently scraped, it will cure them promptly and painlessly. Animals poisoned by this plant have betrayed mischievous congestion of the lungs. By genus it is closely allied to the horned poppy, found so abundantly on our coasts. The tincture of the greater celandine has proved of signal service in small doses for whooping-cough when very spasmodic. In Yorkshire "owl broth" is considered a specific for this complaint. In Gloucestershire a roasted mouse is given to be eaten by the patient. And in Staffordshire the child is made to look at the new moon, whilst the right hand of the nurse is rubbed up and down its bare belly.

